# Unveiling the neural signatures of adolescents with non-suicidal self-injury behavior: an fNIRS study

**DOI:** 10.3389/fpsyt.2025.1604474

**Published:** 2025-07-24

**Authors:** Huishan Liu, Gaizhi Li, Ying Niu, Qiqi Li, Aixia Zhang, Zhifen Liu

**Affiliations:** ^1^ Department of Psychiatry, First Clinical Medical College, Shanxi Medical University, Taiyuan, China; ^2^ Department of Psychiatry, First Hospital of Shanxi Medical University, Taiyuan, China; ^3^ Department of Psychiatry, Shanxi Key Laboratory of Artificial Intelligence Assisted Diagnosis and Treatment for Mental Disorder, First Hospital of Shanxi Medical University, Taiyuan, China

**Keywords:** NSSI, adolescent, fNIRS, DLPFC, candidate neural correlate

## Abstract

**Background:**

Non-suicidal self-injury (NSSI) behavior among adolescents is a significant public health issue, which brings a range of adverse consequences. However, the specific mechanisms underlying this behavior remain unclear. The current study aimed to investigate the hemodynamic activation characteristics of adolescents with NSSI using functional near-infrared spectroscopy (fNIRS) and to explore the correlation of cortical activation with NSSI behavior.

**Methods:**

Eighteen adolescents with NSSI behavior and 24 healthy controls (HCs) participated in this study. Cortical activation (frontal and temporal lobe hemodynamics) were examined using fNIRS. Self-harm frequency, Self-harm functions, and personality traits were assessed by OSI, NSSI - AT, and EPQ respectively.

**Results:**

The Δβ value in the dorsolateral prefrontal cortex (DLPFC) area of the NSSI group was significantly higher than that of the HC group, and the activation level of the DLPFC showed a strong positive correlation with the social communication and the expression subscale of the NSSI-AT.

**Conclusion:**

The specific manifestation of DLPFC cortical activation in adolescents may serve as a candidate neural correlate for NSSI behavior. Targeted improvement of individual social skills and emotional expression abilities is expected to reduce NSSI behaviors.

## 1 Introduction

Non-Suicidal Self-Injury (NSSI) refers to the behavior of directly and deliberately harming bodily tissue (such as cutting or burning) without suicidal intent, which is a significant public health problem (1). A meta-analysis reported that the lifetime prevalence and 12-month prevalence of NSSI among children and adolescents were 22% and 23.2% globally between 2010 and 2021 (2). Deng et al. reported that the NSSI prevalence among adolescents and adults during the COVID-19 pandemic was 32.40% and 15.70% (3). Qu et al. estimated the lifetime prevalence of NSSI among the Chinese youth population to be alarmingly high at 24.7% (4). A number of demographic, personal, and social factors are found to be significantly correlated with NSSI behavior (5). Although NSSI behavior frequently co-exists with conditions such as depression (6), eating disorders, and borderline personality disorder (7), it can also manifest independently (8), such as being linked to emotional instability and compromised social functioning (9); it is also predictive of suicidal tendencies (10). Recognizing its high comorbidity and cross-diagnostic features, NSSI has been proposed as a standalone diagnosis in Section III of the DSM-5 (11). Research has indicated that NSSI behavior is most prevalent among adolescents (7) and is often associated with adverse outcomes such as dropping out of school and difficulties in interpersonal relationships (12), causing immense pain for both the adolescent and their family. Six Specific Psychotherapeutic Interventions (SPI) were found to specifically and significantly reduce NSSI in adolescents: Developmental Group Psychotherapy (DGP), Therapeutic Assessment (TA), the Cutting Down Program (CDP), Emotional Regulation Individual Therapy for Adolescents (ERITA), Treatment for Self-Injurious Behaviors (T-SIB) and Intensive Contextual Treatment (ICT) (13).

In recent years, clinicians and researchers have increasingly emphasized the need for a more multidimensional approach to psychiatric diagnosis and psychopathology research, highlighting the significant heterogeneity within traditional diagnostic (14). Consequently, the search for effective and objective biomarkers has become imperative. However, research exploring the underlying neurobiological systems of NSSI among adolescents has been limited so far. In the past, some studies reported the metabolic alterations in adolescents with major depressive disorder (MDD) exhibiting non-suicidal self-injury (NSSI) using magnetic resonance spectroscopy (MRS), as Zhang suggested that abnormal metabolic changes of N-acetylaspartate (NAA) and choline-containing compounds (Cho) were present in the thalamus in adolescents with depressive disorder and self-injurious behavior (15). Yan et al.’s research identified that elevated Cho and creatine (Cr) levels in the right thalamus might be correlated with increased NSSI risk in male adolescents during major depressive episodes (MDE) (16). However, few studies just focused on NSSI without a depressive episode. Previous functional imaging studies reported specific brain regions associated with Non-Suicidal Self-Injury (NSSI). Some evidence suggests that individuals engaging in self-harm exhibit enhanced activation in the prefrontal regions (including the orbitofrontal cortex and other areas within the prefrontal cortex) during emotional, social, and reward processing (17–19). Findings from previous research have suggested that adolescents engaging in non-suicidal self-injury (NSSI) have significantly reduced gray matter volume in the anterior cingulate cortex (ACC) and multiple prefrontal-limbic brain regions compared to healthy adolescents (20, 21). Additionally, during their resting state, adolescents with NSSI behavior display enhanced connectivity between the amygdala and ACC compared to healthy controls (HC) (22). A study utilizing multimodal magnetic resonance imaging (MRI) revealed significant differences in the temporal gyrus (TG) between Major Depressive Disorder (MDD) patients with a history of NSSI and those without (23). A study using functional near-infrared spectroscopy (fNIRS) also indicated weaker functional connectivity between the prefrontal and temporal lobes in MDD patients with Non-Suicidal Self-Injury (NSSI), as well as abnormal functional connectivity in the frontal lobe associated with NSSI (24). However as interest in multidimensional and cross-diagnostic research continues to grow, so does the need for studies exploring the potential pathophysiological mechanisms underlying NSSI behaviors.

There is a growing need for simple, non-invasive, and reliable scientific techniques to investigate the biological mechanisms of adolescents with NSSI behavior. This would aid in early and accurate diagnosis of the disorder and facilitate the implementation of effective treatment approaches. Functional near infrared spectroscopy (fNIRS) is a non-invasive brain functional imaging technology that indirectly reflects neural activity by monitoring changes in blood flow within the cerebral cortex (25). This technique offers a balance between temporal and spatial resolution, coupled with robust anti-noise capabilities, low cost, high tolerance, and ease of operation (26–28). As such, it is highly suitable as a tool for investigating neural activities in patients with mental illnesses. Further, the Verbal Fluency Task (VFT) is a classic cognitive test commonly used to assess executive functions (29), including inhibitory control, working memory, and cognitive flexibility. It is widely used in fNIRS research to induce activation in the Prefrontal Cortex (PFC) (30). Previous studies have shown that individuals with NSSI have difficulty in inhibitory control and cognitive flexibility (31). Mürner-Lavanchy et al.’s studies have found that during response inhibition tasks, adolescents with NSSI have slightly higher PFC oxygenation than the control group (32).

The primary objective of this study is to investigate the activation of the prefrontal and temporal cortex in adolescents with NSSI and interpret its relationship with NSSI behavior.

## 2 Method

### 2.1 Participants

Between January 2024 and August 2024, 18 adolescent patients with NSSI were recruited from the child and adolescent outpatient departments of the First Hospital of Shanxi Medical University. Inclusion criteria for the patients included the following:

1. Met the criteria of NSSI using the fifth edition of the Diagnostic and Statistical Manual of Mental Disorders (DSM-5) and the Structured Clinical Interview for DSM-5 Research Version (SCID-5-RV) (reporting incidents of NSSI on at least five or more days during the past 12 months);
2. Aged 12–18 years old;
3. No history of any form of psychiatric treatment (including medication, physical and psychological treatment);
4. Right-handedness.

We recruited 24 healthy volunteers from local public schools by sending out e-flyers. The inclusion criteria included: age of 12 to 18 years, with no gender restrictions imposed. We interviewed the HC using M.I.N.I. (MINI-International Neuropsychiatric Interview) to exclude any current or previous mental disorders including NSSI.

The exclusion criteria for both patients and HC subjects included the following:

1. Severe or unstable medical and neurological conditions;
2. Previous diagnoses of schizophrenia or other mental disorders;
3. fNIRS contraindications (open head wounds or acute head trauma, and uncontrolled epileptic seizure episodes).

All the participants were evaluated by two trained child psychiatrists independently.

### 2.2 Clinical evaluation

#### 2.2.1 General demographic data

We collected general demographic data, including gender, age and educational years from all of the participants.

#### 2.2.2 Ottawa self-injury inventory testing

The Ottawa Self-Injury Inventory (OSI) is a self-assessment tool that serves as a comprehensive reporting instrument for evaluating NSSI behavior. It enables a thorough assessment by evaluating the actual frequency of engaging in NSSI behavior (33). Additionally, the questionnaire collects information about the methods and specific anatomical sites used for self-injury. It also explores the role of NSSI behavior in the release of negative emotions, the means through which individuals acquire knowledge about NSSI, strategies employed for preventing NSSI occurrences, and seeking help and treatment after engaging in NSSI behaviors (33). By encompassing these key elements, the OSI enables a comprehensive evaluation of NSSI behavior and related aspects, aiding in a better understanding of the condition and informing interventions. Subjects with suicidal thoughts and behaviors based on question 4 of the OSI were excluded to minimize confounding effects of clinical heterogeneity as much as possible (33).

#### 2.2.3 Non-suicidal self-injury-assessment tool

The NSSI-AT was developed in 2005 to assess primary (such as form, frequency, and function) and secondary (including but not limited to NSSI habituation; contexts in which NSSI is practiced; and NSSI perceived life interference, treatment, and impacts) NSSI characteristics, as well as the complex relationship between NSSI and STB (34). For those who screen positive to the initial NSSI assessment, the NSSI-AT generally requires from 5 to 20 min, depending on modules used and the degree of detail respondents provide in qualitative fields. The Chinese version of NSSI-AT Function part was translated by Zhang et al (35). In the current study, we adopted the function part of NSSI-AT, including the Self-retribution and deterrence, Affective imbalance-low pressure, Affective imbalance-high pressure, Social communication and expression, and Sensation seeking subscales.

#### 2.2.4 Eysenck personality questionnaire

In this study, the Chinese-revised version of the Eysenck Personality Questionnaire (EPQ) for children was employed to assess the personalities of the participants (36). This questionnaire comprises 88 true-false items and encompasses four subscales: Extroversion (E), Psychoticism (P), Neuroticism (N), and Lie (L). The overall scale exhibits a Cronbach’s alpha coefficient of 0.70, while the Cronbach’s alpha coefficients for the E, P, N, and L subscales are 0.76, 0.76, 0.88, and 0.77, respectively. Based on the raw scores obtained by participants on each subscale, standard T scores (T = 50 + 10 × (x − m)/SD) can be converted from norms to analyze their personality characteristics. Within each subscale, T scores ranging from 43.3 to 56.7 points are considered intermediate; T scores between 38.5 and 43.3 points or between 56.7 and 61.5 points are indicative of a propensity type; and T scores below 38.5 points or above 61.5 points are deemed typical (36).

### 2.3 Functional near-infrared spectroscopy

**Figure 1:**
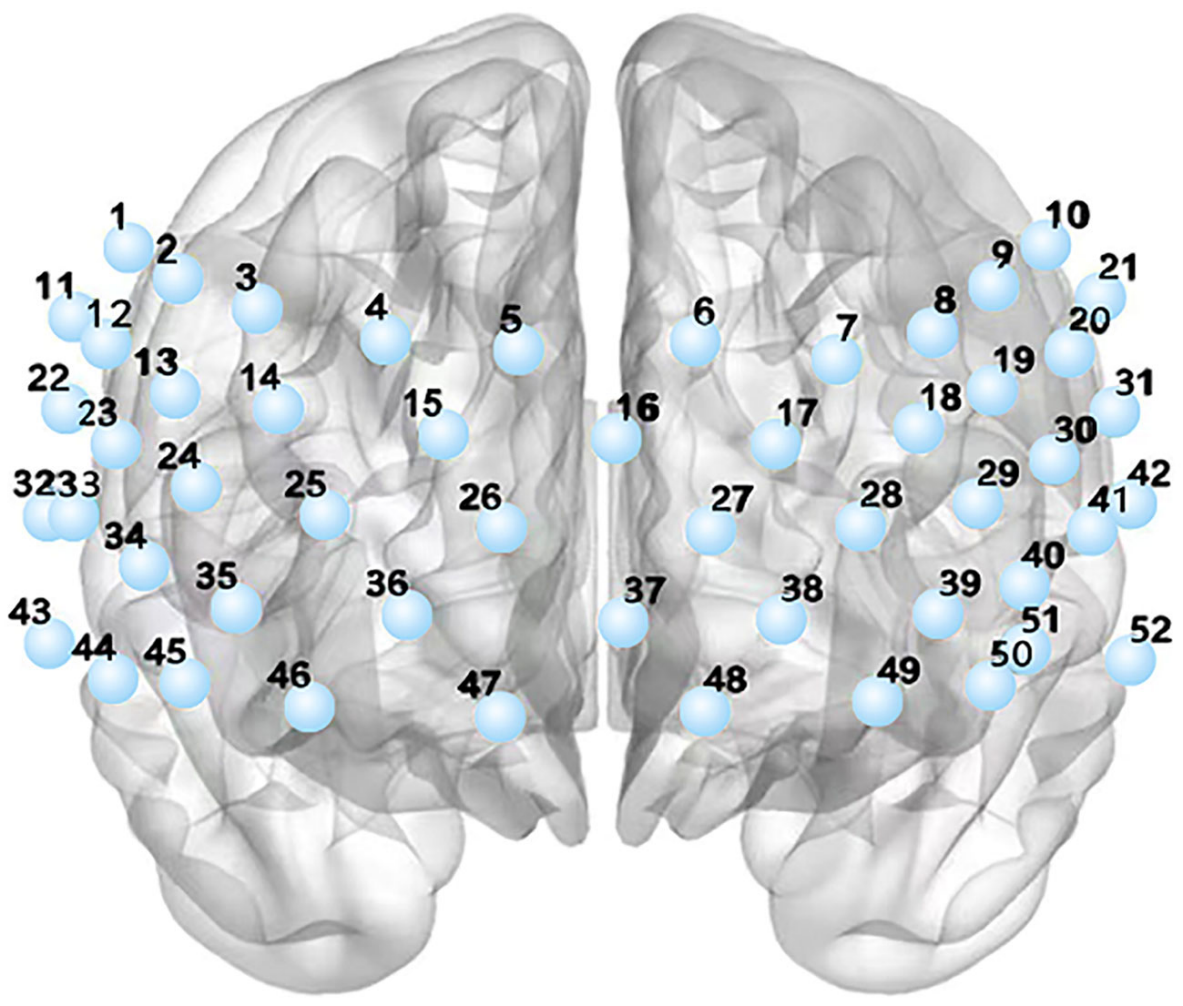
The distribution of 52 channels.

This study utilized a 52-channel functional near-infrared spectroscopy (fNIRS) system equipped with 17 emitters and 13 receivers (ETG-4100, Hitachi Medical Co., Tokyo, Japan) to measure the hemodynamic responses in the prefrontal and superior temporal cortices. By emitting near-infrared light at wavelengths of 690 nm and 830 nm, the concentration changes of oxyhemoglobin (HBO) were determined. The time resolution of the fNIRS data was set at 10 Hz. See **Figure 1**.

### 2.4 Regions of interest

To analyze the cortical activation, each channel was projected onto the cortical surface using the NIRS-SPM (37) based on the Automated Anatomical Labeling (AAL) (38) template. Subsequently, the 52 channels were divided into six regions of interest (ROIs) based on the maximum overlap probability: Dorsolateral prefrontal cortex area (detected by the 7th, 14th, 18th, 25th, 35th, 39th, 45th and 50th channels), Frontopolar area (detected by the 15th, 16th, 17th, 25th, 26th, 27th, 28th and 37th channels), Subcentral area (detected by the 22th, 31th and 33th channels), Orbitofrontal area (detected by the 36th, 38th, 46th, 47th, 48th and 49th channels), Middle Temporal gyrus area (detected by the 52th, 43th and 44th channels) and pars triangularis Broca’s area (detected by the 13th, 19th, 24th, 29th, 34th and 40th channels).

### 2.5 Verbal fluency test

**Figure 2:**
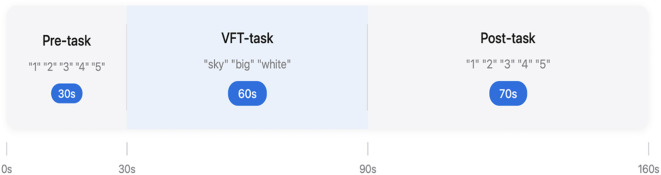
Verbal fluency test (VFT).

The Verbal Fluency Test (VFT) task was adapted to examine the cortical activation of the participants. The VFT test comprises a 30-second pre-task rest period, a 60-second VFT task, and a 70-second post-task rest period. During the VFT task, the participants were asked to combine words containing specific Chinese characters (“sky”(Chinese character “tian”),”big”(Chinese character “da”),”white”(Chinese character “bai”)), the instruction is as follows “please combine words using “tian, da, bai”, using at least three words, the more words, the better. See **Figure 2**.

### 2.6 fNIRS statistical analysis

The data of HBO collected by fNIRS were processed using MATLAB 2013b and the NIRS-SPM toolbox. (1) Preprocessing: The raw data were processed through a hemodynamic response function (HRF) and discrete cosine transform (DCT) to eliminate noise such as drifts and artifacts (e.g., head movements). We also performed bad channel removal. In this step, channels with NaN (Not-a-Number) beta values calculated by NIRS-SPM were identified as invalid channels. These bad channels were excluded from the calculation of average beta values within regions of interest (ROI). The bandpass filtering range we applied was 0.01–0.10 Hz. (2) Construction of General Linear Model (GLM): A GLM equation Y = βX + ϵ was established, where Y represents the changes in HBO detected by fNIRS, X represents the predicted values derived from the experimental design, and ϵ denotes the unexplained error. β is the fitting coefficient, representing the level of cortical activation induced by VFT in this study. (3) Calculation of Δβ values for HBO, which is the β value during the VFT task minus the baseline β value. The Δβ value represents the level of cortical activation in the prefrontal and temporal lobes during the VFT task.

### 2.7 Data analysis

Demographic data was analyzed by SPSS 22.0. Statistical analyses of fNIRS data were performed using the GRETNA-2.0 toolkit. Independent sample t-tests were conducted between the NSSI group and the healthy control group, with age, gender, and educational years as covariates. When analyzing the correlation between Δβ values and clinical symptoms, partial correlation analyses were performed with age, gender, and years of education as covariates. All of the tests were two-tailed. The p-values were corrected for multiple comparisons using the False Discovery Rate (FDR), and P(FDR) < 0.05 was considered statistically significant.

## 3 Results

### 3.1 Demographic data and clinical characteristics

Results indicated that the age and years of education in the NSSI group were significantly lower than those in the HC group (p < 0.001), and a significant difference was not observed within gender between the two groups (p > 0.05). The NSSI adolescents scored significantly higher on the EPQ-P (p = 0.041) and EPQ-N (p < 0.001) subscales compared to the healthy control group, while the scores on the EPQ-E (p < 0.001) and EPQ-L (p = 0.001) subscales were significantly lower than those of the healthy control group. See **Table 1**.

**Table 1:** Demographic data and clinical data between the two groups.

Variables		NSSI group (n=18)	HC group (n=24)	t/*x* ^2^	P
Age		14.50 ± 1.54	17.29 ± 0.62	-7.242	<0.001
Gender		4:14	2:22	1.620	0.375
Educational years		9.00 ± 1.78	11.00 ± 0.00	-4.761	<0.001
OSI	Age of first NSSI	12.44 ± 2.22	–		
	NSSI frequency (1 week)	2.16 ± 2.40	–		
	NSSI frequency (1 month)	10.16 ± 10.69	–		
	NSSI frequency (6 month)	42.61 ± 53.00	–		
	NSSI frequency (12 month)	63.61 ± 75.86	–		
NSSI-AT	Self-retribution and deterrence	5.33 ± 2.72	–		
	Affective imbalance-low pressure	7.72 ± 2.19	–		
	Affective imbalance-high pressure	5.50 ± 2.38	–		
	Social communication and expression	1.22 ± 1.66	–		
	Sensation seeking	6.16 ± 3.05	–		
	Social dimensions of NSSI practice	5.61 ± 3.32	–		
	Routines	5.94 ± 3.81	–		
EPQ	EPQ-P	57.19 ± 7.93	50.83 ± 9.40	2.124	0.041
	EPQ-E	28.86 ± 12.32	55.00 ± 9.89	-7.174	<0.001
	EPQ-N	72.99 ± 5.25	48.95 ± 13.34	6.422	<0.001
	EPQ-L	35.17 ± 10.21	46.87 ± 5.27	-3.988	0.001

*p < 0.05; **p < 0.01.

### 3.2 Δβ in HBO during the VFT

**Figure 3:**
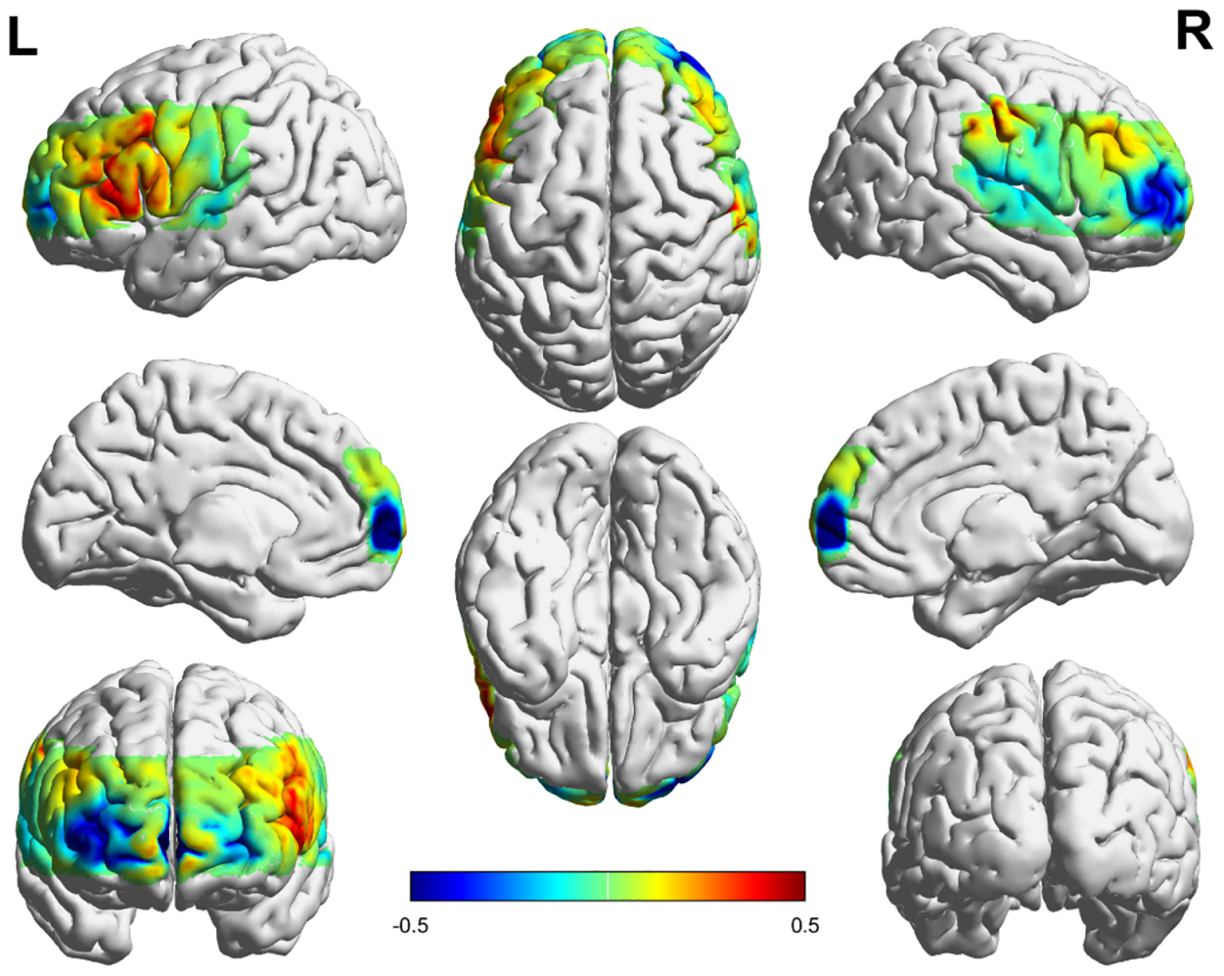
Activation in the frontal and temporal lobes in the NSSI group. The numerical value represents the average β value of the NSSI group, Positive values indicate positive activation, Negative values indicate negative activation.

**Figure 4:**
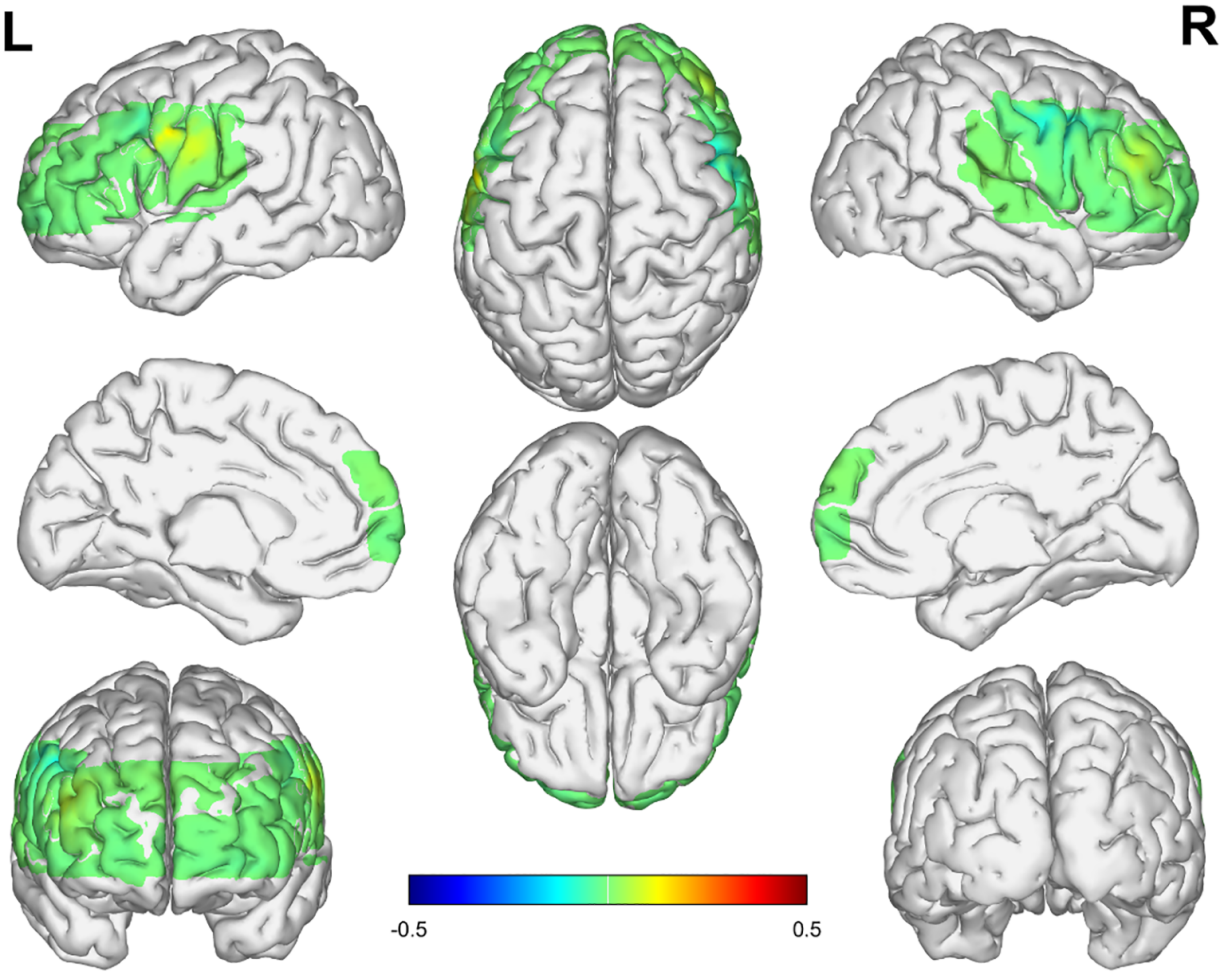
Activation in the frontal and temporal lobes in the HC group. The numerical value represents the average β value of the HC group, Positive values indicate positive activation, Negative values indicate negative activation.

**Figure 5:**
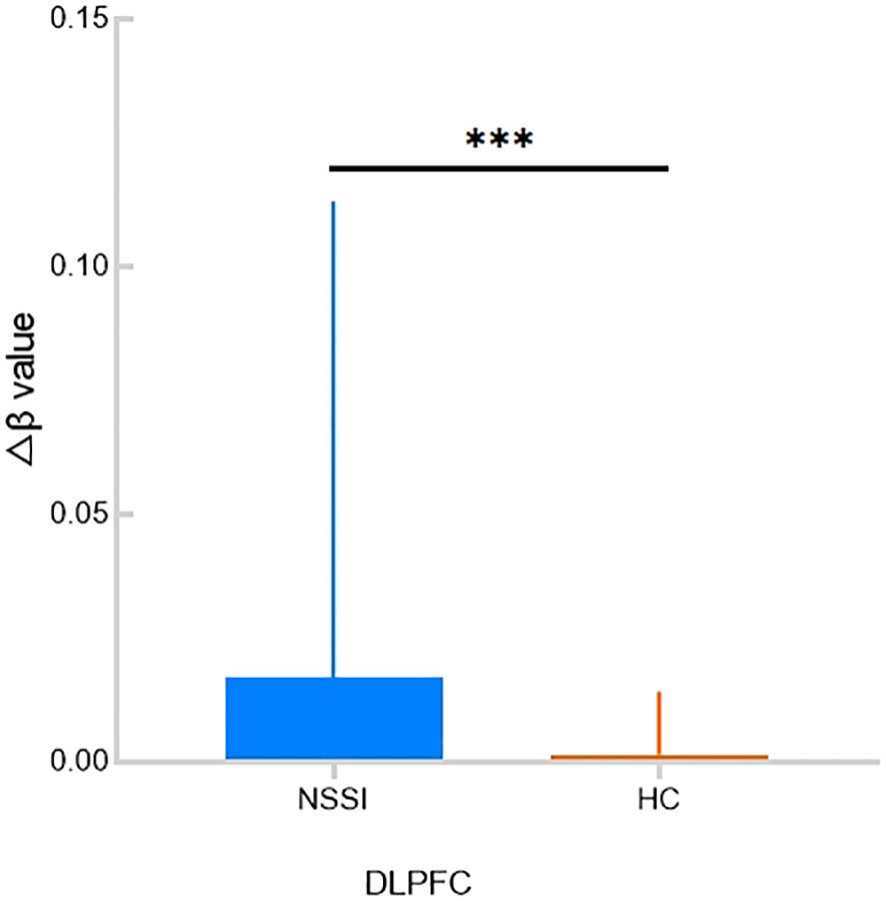
The Δβ value of DLPFC between the two groups. ***indicates p < 0.001.

The he Δβ values in the dorsolateral prefrontal cortex (DLPFC) of the NSSI group were significantly higher than those in the HC group (p = 0.0007). No significant differences in Δβ values were observed in the remaining areas. See **Table 2**, **Figures 3**–**5**.

**Table 2:** The comparison of Δβ between the two groups.

Region	NSSI group (n=18)	HC group (n=24)	P-value	P-value (FDR correction)	T-value
Dorsolateral prefrontal cortex	0.0172	0.0016	0.0007	0.0049*	3.6979
Frontopolar area	-0.1422	-0.0035	0.7150	0.83417	-0.3680
Subcentral area	-0.1512	0.0052	0.2517	0.44047	1.1645
Orbitofrontal area	0.1430	-0.0140	0.1925	0.44047	1.3275
Middle Temporal gyrus	-0.1371	0.0036	0.9300	0.93	-0.0884
pars triangularis Broca’s area	0.2674	0.0130	0.0168	0.0588	2.5056

*p < 0.05.

### 3.3 Correlation of clinical symptoms and Δβ values in the DLPFC

**Figure 6:**
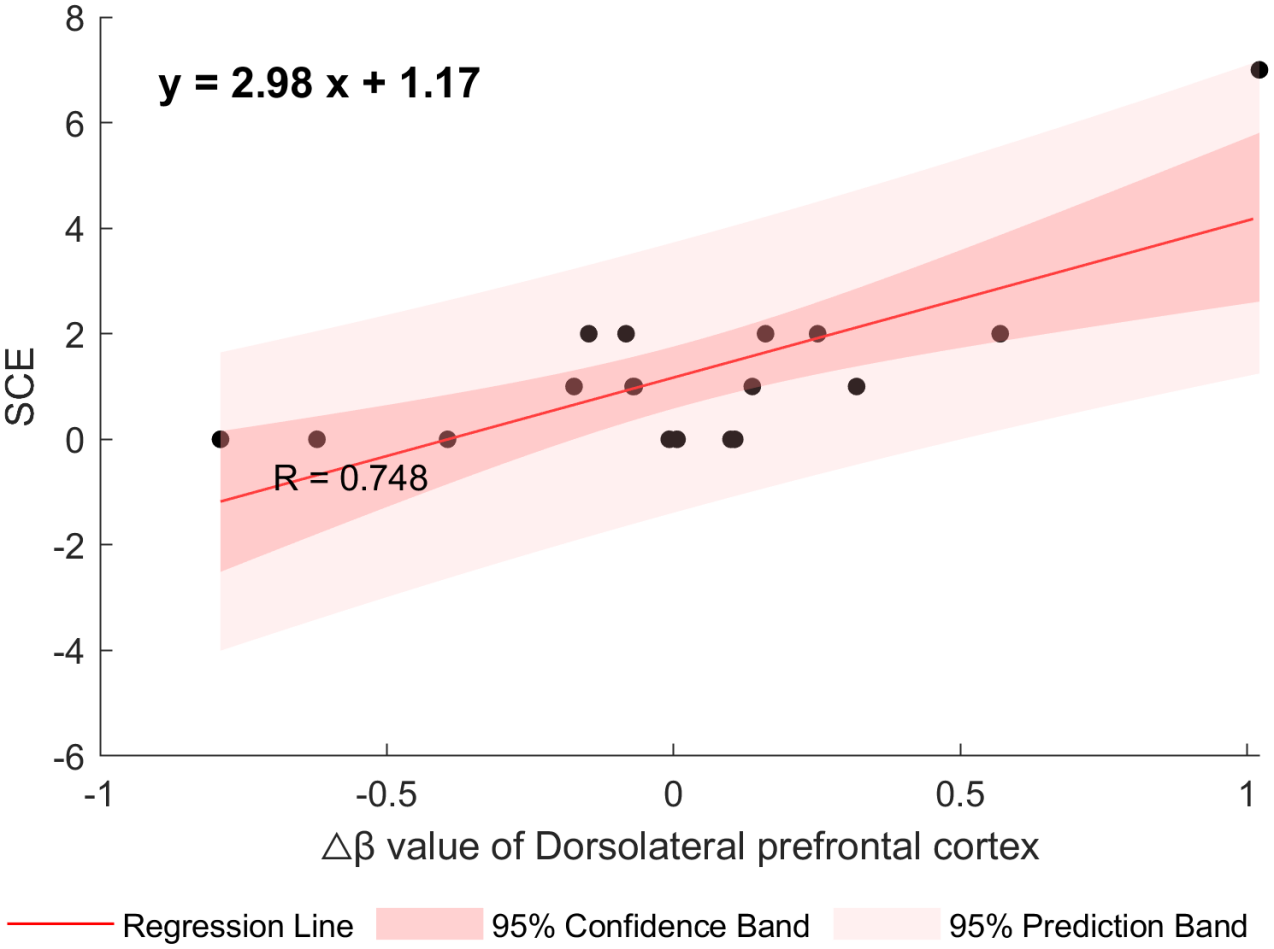
The correlation between Δ β of DLPFC and social communication and expression (SCE).

After controlling for age, gender, and years of education, a partial correlation analysis was conducted between clinical symptom-related scales and the Δβ values in the DLPFC area. The results showed that the Δβ values in the DLPFC of adolescents with NSSI were significantly positively correlated with the Social Communication and Expression Subscale (r = 0.7483, p = 0.0013), corrected using FDR correction. (See **Table 3**, **Figure 6**).

**Table 3:** The correlation of Δβ of DLPFC and clinical variables.

DLPFC	*r*	*P (FDR correction)*
Age of first NSSI	-0.0873	0.7572
NSSI frequency (1 week)	-0.1929	0.4909
NSSI frequency (1 month)	-0.3281	0.2326
NSSI frequency (6 month)	-0.2848	0.3035
NSSI frequency (12 month)	-0.2033	0.4674
Affective imbalance-low pressure	0.1371	0.626
Affective imbalance-high pressure	0.0106	0.9702
Social communication and expression	0.7483	0.0013*
Self-retribution and deterrence	-0.0853	0.7624
Sensation seeking	0.1298	0.6449
Social dimensions of NSSI practice	0.4846	0.0671
Routines	-0.2717	0.3273
EPQ-P	0.1477	0.6648
EPQ-E	0.3784	0.2511
EPQ-N	0.1527	0.6541
EPQ-L	-0.044	0.8977

*p < 0.05.

## 4 Discussions

Our study aimed to investigated the differences of cortical activation between adolescents with NSSI and a healthy control group during VFT using fNIRS. Furthermore, we examined whether the task-based fNIRS activity correlated with NSSI behavior. The results of the study revealed that the degree of cortical activation in the dorsolateral prefrontal cortex (DLPFC) was significantly higher in the NSSI group compared to the healthy control group, and the level of DLPFC activation was strongly positively correlated with the social communication and expression subscales. This study adds to the evidence regarding the candidate neural correlates of NSSI in adolescents.

Results indicate distinct personality patterns in adolescents with NSSI based on the Eysenck Personality Questionnaire (EPQ). These adolescents exhibit obvious emotional instability, frequently experiencing intense emotions and overreacting to stimuli. This makes it difficult for them to restore emotional balance after arousal. Additionally, compared with healthy controls, the NSSI group has slightly higher scores on the psychoticism subscale. This indicates slightly higher levels of aggressive tendencies, oppositional feelings, and reduced adaptability. Furthermore, these adolescents tend to be introverted, preferring solitude over social interaction, which may exacerbate feelings of loneliness and emotional distress. These findings are consistent with recent studies. Kang et al. (2021) identified comparable patterns of emotional instability and introversion in college students with NSSI, emphasizing that emotional instability is central to maintaining self-injurious tendencies (39). Building on these findings, Peng et al. (2022) observed similar traits in adults with depressive disorders and NSSI (40). The study noted that patients with MDD and NSSI presented higher levels of EPQ-psychoticism and more immature defense styles compared to healthy controls (40). This reduced defensive capacity may be attributed to developmental factors, such as age-related differences in emotional regulation and varying degrees of socialization, which could impair the NSSI group’s ability to cope with interpersonal stress effectively (41). Collectively, these findings highlight the importance of integrating emotional regulation training and social skills interventions into therapeutic approaches for people with NSSI, particularly to address their heightened emotional instability and introverted tendencies.

Further, NSSI is often triggered by emotional outbursts (42), and individuals with NSSI consistently report poorer emotion regulation abilities compared to their non-self-injuring peers (43). Studies have demonstrated a mutual influence between NSSI and emotion regulation (44). Individuals with NSSI tend to use cognitive reappraisal strategies less frequently and use more emotional suppression strategies (45).

The DLPFC is a region highly associated with cognitive control, emotional regulation, expression, and response inhibition (46–48).Functional magnetic resonance imaging (fMRI) studies indicate that downregulation of negative emotions under cognitive reappraisal conditions is associated with stronger activation of the DLPFC (49). Our study found that adolescents with NSSI exhibited increased cortical activation in the DLPFC compared to the healthy control group, which is consistent with previous fMRI Mürner-Lavanchy et al., demonstrated that adolescents with non-suicidal self-injury (NSSI) exhibit marginally higher prefrontal cortex oxygenation levels during inhibitory control tasks compared to healthy controls (32), however, Koenig et al. (50)observed significantly decreased PFC oxygenation during resting state, which is different from our study; they proposed that activity of the PFC might switch from under-(adolescence) to over- (adult) activation as a function of age and development. Importantly, most of the existing studies have been conducted in relatively small samples and most studies are task-based. Furthermore, in MDD patients with NSSI behaviors, weaker functional connectivity was observed between the left dorsolateral prefrontal cortex and left premotor cortex (LDLPFC-LPMC) relative to healthy populations (24).

No significant between-group differences were observed in other ROIs (including Frontopolar area, Subcentral area, Orbitofrontal area, Middle Temporal gyrus area and pars triangularis Broca’s area), except for the DLPFC. This suggests that NSSI behavior may have specific functional impairments in the dorsolateral prefrontal cortex. Most of the fNIRS studies focused on the DLPFC, and while current fMRI studies used task- based fMRI approach, they mainly investigated the networks or connectivity of the amygdala. For example, Nam et al. demonstrated that the NSSI group had increased activity relative to the control group in the inferior parietal lobe, inferior temporal gyrus, calcarine, insula, and thalamus in response to positive self-referential stimuli. They also showed greater activation in the calcarine and reduced activation in the inferior frontal gyrus in response to negative self-referential stimuli compared with the control group (51). Ho et al. found that diminished coherence between the default mode and salience networks and higher connectivity between the central executive and default-mode networks (52). Factors such as task difficulty, complexity, and presentation methods can all influence the activation levels of brain regions. In the future, it is necessary to combine multiple paradigms or multimodal imaging to further validate the results.

We did not observe a direct correlation between cortical activation in the DLPFC and the frequency of NSSI. Few studies focused on the presence or frequency of NSSI and cortical activation. Zahid et al. observed that participants who engaged in NSSI exhibited some deactivation of the DLPFC when faced with more difficult cognitive challenges (53). These findings suggest that NSSI behavior is an intricate behavior associated with multiple psychological, social, and biological factors. It is not solely attributable to the activation of a single brain region but rather the consequence of intricate interactions among multiple systems and networks. Moving forward, we could consider incorporating mediating or moderating variables into studies examining the frequency and severity of NSSI among adolescents.

Furthermore, within the NSSI group, we identified a robust positive correlation between cortical activation in the dorsolateral prefrontal cortex (DLPFC) and social communication and expression score of NSSI-AT. This underscores the DLPFC’s pivotal role in facilitating social interaction and emotional expression. Given that social communication and expression critically influence interpersonal relationships, and considering the integrated theoretical model of NSSI which categorizes risk factors into interpersonal and intrapersonal domains (54), this finding holds significant relevance. Interpersonal risk factors in adolescents may motivate or trigger NSSI engagement (55), while intrapersonal risk factors can increase vulnerability to stress coping via emotional dysregulation (56), potentially leading to NSSI. Although adolescents with NSSI often behaviorally avoid social interaction, they paradoxically seek social support, attention, or assistance (57), reflecting a defense mechanism of reaction formation and indicating an underlying need to be understood and to establish healthy interpersonal relationships. Successful social interaction necessitates continuous environmental assessment, interpretation of others’ emotions and behaviors, and generation of appropriate responses. The observed DLPFC activation likely reflects the neural demand associated with processing this complex social information to facilitate engagement. This correlation suggests that while NSSI behavior may not be directly linked to the degree of DLPFC activation, it could be associated with deficits in social competence, emotional expression, or emotion regulation strategies. Consequently, this insight offers potential avenues for NSSI intervention and treatment by targeting the enhancement of social skills and emotional expression capabilities, thereby potentially reducing the occurrence of NSSI behaviors.

## 5 Limitation

There are several limitations in the current study. First, the sample size of adolescents with NSSI is relatively small. Secondly, the age and educational years between the two groups were shown to be statistically different, thus we added these data as covariates when analyzed. However, this preliminary study is the first study examining the cortical activation in Chinese adolescents. Given that adolescence represents a critical period for DLPFC development, age disparities may contribute to the observed group differences. We will continue to include adolescents with NSSI and also recruit healthy controls who are matched in terms of demographic data to validate our preliminary results.

## 6 Conclusion

The specific manifestation of DLPFC cortical activation in adolescents with NSSI may serve as a candidate neural correlate for NSSI behavior. Targeted improvement of individual social skills and emotional expression abilities is expected to reduce NSSI behaviors. This discovery not only confirms the link between NSSI behavior and brain function abnormalities but also provides new insights and evidence for understanding the underlying neural mechanisms of NSSI in adolescents, laying a theoretical foundation for further clinical interventions and treatment strategies.

## Data Availability

The raw data supporting the conclusions of this article will be made available by the authors, without undue reservation.
